# Biomimetic anti-inflammatory and osteogenic nanoparticles self-assembled with mineral ions and tannic acid for tissue engineering

**DOI:** 10.1186/s40580-022-00338-2

**Published:** 2022-10-10

**Authors:** Hayeon Byun, Gyu Nam Jang, Min-Ho Hong, Jiwon Yeo, Hyunjung Shin, Won Jong Kim, Heungsoo Shin

**Affiliations:** 1 Department of Bioengineering, Hanyang University, 222 Wangsimni-ro, Seongdong-gu, Seoul, 04763 Republic of Korea; 2 BK21 FOUR Education and Research Group for Biopharmaceutical Innovation Leader, Department of Bioengineering, Hanyang University, 222 Wangsimni-ro, Seongdong-gu, Seoul, 04763 Republic of Korea; 3 Department of Dental Biomaterials and Research Institute of Oral Science, College of Dentistry, Gangneung-Wonju National University, Gangneung, 25457 Republic of Korea; 4 Department of Chemistry, Pohang University of Science and Technology (POSTECH), Pohang, 37673 Republic of Korea; 5 Nature Inspired Materials Processing Research Center, Department of Energy Science, Sungkyunkwan University, Suwon, 16419 Republic of Korea; 6 Institute of Nano Science and Technology, Hanyang University, 222 Wangsimni-ro, Seongdong-gu, Seoul, 04763 Republic of Korea

**Keywords:** Multi-functional biomaterial, Supramolecular self-assembly, Metal phenolic network, Tannic acid, Mineral nanoparticle, Anti-inflammation, Tissue engineering

## Abstract

Bone healing involves complex processes including inflammation, induction, and remodeling. In this context, anti-inflammatory and osteoconductive multi-functional nanoparticles have attracted considerable attention for application in improved bone tissue regeneration. In particular, nanoparticles that promote suppression of inflammatory response after injury and direction of desirable tissue regeneration events are of immense interest to researchers. We herein report a one-step method to prepare multi-functional nanoparticles using tannic acid (TA) and simulated body fluid (SBF) containing multiple mineral ions. Mineral-tannic acid nanoparticles (mTNs) were rapidly fabricated in 10 min, and their size (around 250–350 nm) and chemical composition were controlled through the TA concentration. In vitro analysis using human adipose derived stem cells (hADSCs) showed that mTNs effectively scavenged reactive oxygen species (ROS) and enhanced osteogenesis of hADSCs by inducing secretion of alkaline phosphatase. mTNs also increased osteogenic marker gene expression even in the presence of ROS, which can generally arrest osteogenesis (*OPN*: 1.74, *RUNX2*: 1.90, *OCN*: 1.47-fold changes relative to cells not treated with mTNs). In vivo analysis using a mouse peritonitis model revealed that mTNs showed anti-inflammatory effects by decreasing levels of pro-inflammatory cytokines in blood (IL-6: 73 ± 4, TNF-α: 42 ± 2%) and peritoneal fluid (IL-6: 78 ± 2, TNF-α: 21 ± 6%). We believe that this one-step method for fabrication of multi-functional nanoparticles has considerable potential in tissue engineering approaches that require control of complex microenvironments, as required for tissue regeneration.

**Supplementary Information:**

The online version contains supplementary material available at 10.1186/s40580-022-00338-2.

## Introduction

Polyphenols are naturally occurring plant-derived organic compounds with multiple phenol units. Polyphenols have been widely utilized in biomedical applications because of their unique biological and chemical properties [1–3]. The hydroxyl groups in these molecules act as proton and electron donors that can scavenge reactive oxygen species (ROS), and polyphenols exhibit anti-inflammatory, anti-bacterial, anti-cancer, anti-allergic, and anti-diabetes properties [4–6]. Chemically, polyphenols show strong affinity to biomolecules such as proteins and ions [7]. For example, metal phenolic networks (MPNs) are readily formed with various cations such as Ca^2+^, Fe^3+^, and Cu^2+^ via metal–ligand coordination reactions, and these reactions have been used to deposit polyphenols on the surfaces of biomaterials [8, 9]. Catechin, tannic acid (TA), and epigallocatechin gallate (EGCG) have been used for universal coating of biomaterials independent of their surface chemistry through oxidation and MPN formation of polyphenols [10]. For example, Payra et al. confirmed that TA coating of metal alloys improved the surface hydrophilicity and anti-bacterial properties [11], while Lee et al. demonstrated that catechin-coating of polycaprolactone substrate enhanced osteogenesis of human stem cells [12]. Furthermore, through self-assembly of polyphenols with therapeutic agents, they can be used as drug delivery systems, leading to particulate or capsule formation [13].

Among various polyphenol molecules, TA has received particular attention as it possesses several aromatic rings and hydroxyl groups [14]. TA interacts strongly with biological molecules such as polymers, proteins, and ions through hydrogen bond formation, electrostatic interactions, and metal coordination, which has led to the use of TA for material engineering purposes such as surface modification and particle assembly [15]. In addition, the presence of abundant amphiphilic pyrogallol groups in TA is advantageous for colloidal nanoparticle production [16]. TA-based nanoparticle fabrication has been accomplished through supramolecular self-assembly, which involves the formation of individual nanoparticles via repulsion between particles at the same time as the growth of monomers by burst nucleation [17]. The process is achieved primarily through the coordinated assembly of metal ions and TAs, and the dimension and chemical structure of nanoparticles can be controlled by varying the concentrations and types of metal ions [18]. For example, Phiwchai et al. controlled particle size and cell internalization by changing the ratio of Fe ions and TA [19]. Nevertheless, the phenol-rich chemical structure of TA sometimes interferes with the formation of nanoparticles; self-assembly of TA itself without polymeric templates is challenging given the high solubility of TA, the difficulty of controlling the size and composition of nanoparticles owing to rapid chemical reactions, and the generation of impurities from MPN-based nanomaterials depending on solvent type [15, 20, 21]. Furthermore, the majority of previous studies have used single ionic solutions for nanoparticle formation, which can result in instability on hydrophilic substrates.

Mineral ions such as cobalt, copper, and calcium are essential chemical elements for cellular activity as they serve as cofactors for enzymes and have metabolic functions such as activation of ion channels [22, 23]. These metal ions have been incorporated into single metals or alloy biomaterials to control their local concentrations and then used in tissue regeneration [23, 24]. For example, borate was doped onto bioactive glass microparticles to promote angiogenesis [25]. Kulanthaive et al. used hydroxyapatite doped with cobalt and magnesium to increase osteogenic differentiation and angiogenesis of pre-osteoblasts [26]. It should be noted that more than 60% of bone tissue comprises inorganic compounds and thus, mineral-based biomaterials such as hydroxyapatite, nanoclay, and β-tricalcium phosphate have been widely used for bone tissue engineering [27, 28]. Mineralization of the surfaces of materials via calcium phosphate precipitation using simulated body fluid (SBF) has also been actively studied. Mineralization is advantageous for incorporating biomolecules that are sensitive to external conditions such as temperature, pH, and pressure thanks to physiological reaction conditions [29]. Furthermore, minerals formed by this method exhibit crystallinity and a dissolution rate similar to that of bone minerals, which can assist in bone formation [30]. However, several limitations such as long processing times and risk of chronic inflammation have been reported [29, 31, 32].

In this study, we studied one-step nanoparticle formation through supramolecular self-assembly of TA in an ion-rich environment containing multiple mineral ions with the overall goal of developing ROS scavenging, anti-inflammatory, and osteoconductive multi-functional nanoparticles suitable for use as bone tissue regeneration materials. We hypothesized that nanoparticles would be rapidly and stably produced by mixing TA and SBF as well as that the size and chemical composition of the nanoparticles could be controlled by changing the TA concentration. We investigated the biological activity of nanoparticles through in vitro tests using human adipose derived stem cells (hADSCs) and in vivo tests in a mouse peritoneal model.

## Methods/experimental

### Materials

Sodium chloride (NaCl), magnesium chloride (MgCl_2_), and sodium hydroxide (NaOH) were purchased from Junsei (Tokyo, Japan). Calcium chloride (CaCl_2_) was obtained from DUKSAN (Kyungki-do, Korea). Sodium bicarbonate (NaHCO_3_), potassium chloride (KCl), potassium bromide (KBr), sodium phosphate dibasic (Na_2_HPO_4_), silver nitrate, sodium thiosulfate, formalin solution, Folin–Ciocalteu reagent, sodium carbonate (Na_2_CO_3_), tannic acid (TA), thiazolyl blue tetrazolium bromide (MTT), ascorbic acid, 3% hydrogen peroxide (H_2_O_2_), iron (III) chloride hexahydrate, 1,10-phenanthroline, 2,2′-azino-bis(3-ethylbenzothiazoline-6-sulfonic acid) diammonium salt (ABTS), alkaline phosphatase yellow (pNPP) liquid, alizarin red S, 2′,7′-dichlorofluorescein diacetate (DCF-DA), dimethyl sulfoxide, cetylpyridinium chloride, and Zymosan A from *Saccharomyces cerevisiae* were purchased from Sigma Aldrich (St. Louis, MO, USA). QuantiChrom™ Calcium assay kit was purchased from Bioassay Systems (Hayward, CA, USA). Phosphate-buffered saline (PBS) and Dulbecco’s phosphate buffered saline (DPBS) were purchased from Welgene (Gyeongsan-si, Korea). Penicillin–streptomycin (PS) and trypsin/EDTA (TE) were purchased from Wisent (St. Bruno, QC, Canada). Dulbecco’s modified Eagle’s medium (DMEM), MesenPRO RS™ medium, and fetal bovine serum (FBS) were purchased from Gibco BRL (Carlsbad, CA, USA). StemPro™ hADSCs, LIVE/DEAD assay kit, and Alexa Fluor™ 488 Phalloidin were purchased from Invitrogen (Carlsbad, CA, USA). Maxime RT Premix was purchased from Intron (Seoul, Korea). SYBR Premix Ex Taq was acquired from TAKARA (Otsu, Shiga, Japan). ELISA kits for IL-6 and TNF-α were purchased from Koma Biotech (Seoul, Korea).

### Fabrication and characterization of mTNs

Mineral-tannic acid nanoparticles (mTNs) were prepared by self-assembly of TA with mineral ions in 10 × SBF solution (58.43 g NaCl, 3.6754 g CaCl_2_, 0.3538 g KCl, 1.016 g MgCl_2_, and 1.4 g Na_2_HPO_4_ in 1 L of distilled water (DW), pH 4.35) and various concentrations of TA (0.5, 5, and 10 mg/ml). First, TA was dissolved in 10 × SBF solution, and the pH of the solution was adjusted to 4.35. Then, NaHCO_3_ was added to the solution at a final concentration of 0.04 M and stirred at 650 rpm for 10 min at room temperature (RT). mTNs were collected via centrifugation (4000 rpm for 5 min) and washed with DW several times. Then, the mTNs were re-dispersed in Tris–HCl buffer (pH 8.8) and incubated for 10 min at RT for stabilization of the particles via deprotonation. Finally, the mTNs were washed and lyophilized prior to use. Surface morphology of mTNs was observed using field emission scanning electron microscopy (FE SEM; JSM 7600F, JEOL, Tokyo, Japan), and the size of mTNs was measured using image J software (NIH). mTNs were dispersed by a Sonifier^®^ (BRANSON, St. Louis, USA) in DW, and absorbance was measured at 280 nm using a microplate reader (Varioskan LUX, Thermo Scientific, Waltham, MA, USA). Total phenol content in mTNs was quantified by Folin–Ciocalteu assay. For this assay, mTNs were dispersed in DW, the same volume of Folin–Ciocalteu reagent was added and allowed to react for 10 min, and then 600 μl of 2% sodium carbonate was added followed by a 1-h incubation at RT. After incubation, the absorbance of the solution was measured at 760 nm via a microplate reader. The calcium content of mTNs was measured using the QuantiChrom™ calcium assay kit (BioAssay Systems, Hayward, CA, USA). mTNs were dissolved in 0.6 N HCl overnight at 37 °C and reacted with calcium assay reagent for 3 min. After the reaction, absorbance was measured at 612 nm via microplate reader. We measured the total weight of self-assembled mTNs to investigate the effect of TA in the self-assembly process. The crystallographic phase of mTNs was analyzed by x-ray diffraction (XRD; SmartLab, Rigaku, Tokyo, Japan), and results were interpreted using JADE software (Christchurch, New Zealand) and the International Center for Diffraction Data (ICDD) database. mTNs were reacted with KBr to create pellets for analysis of surface chemistry of particles via Fourier transform infrared spectroscopy (FT-IR; Nicolet 6700, Thermo Fisher Scientific, Waltham, UK). The elemental composition of mTNs was analyzed via x-ray photoelectron spectroscopy (XPS; ESCALAB 250Xi, Thermo Fisher Scientific, Waltham, UK). XPS high-resolution scans were performed for the C1s, O1s, Ca2p, and P2p core levels.

### Cytotoxicity and ROS scavenging activity of nanoparticles

We used mTNs prepared with 10 mg/ml of TA for cell cytotoxicity and ROS scavenging assays. hADSCs (passage number < 7) were used for these experiments and cultured under standard conditions (5% CO_2_, 37 °C). Cells were cultured in MesenPRO RS™ medium supplemented with 1% PS. To assess the cytotoxicity of TA and mTNs, 10,000 hADSCs were seeded on a 24 well-tissue culture plate and cultured for 24 h under standard conditions. Transwell^®^ (Corning, Lowell, MA, USA) inserts were placed on the plate, following which TA and mTNs at various concentrations were added (0, 2, 10, and 50 μg/ml). After 3 days of treatment, LIVE/DEAD staining was performed to assess cell viability, and the samples were observed via fluorescence microscopy (TE 2000; Nikon, Tokyo, Japan). To quantify the viability of the cells, MTT solution was added to the cells followed by a 1-h incubation; the MTT solution was then removed, and DMSO was added to dissolve the MTT formazan crystals. Absorbance was measured at 550 nm via a microplate reader. For the Fe conversion test, 1, 10-phenanthroline solution was prepared by mixing 1 mg/ml of 1, 10-phenanthroline and 1 mM of FeCl_3_ dissolved in DW. Then mTNs at various concentrations (0, 2, 10, and 50 μg/ml) were dispersed in the solution followed by a 30-min incubation at RT. After the reaction, the absorbance of the solution was measured at 510 nm using a microplate reader. To assess the ABTS inhibition capacity of mTNs, ABTS solution was prepared by dissolving ABTS (7.0 mM) and potassium persulfate (2.4 mM) in DW and the solution was diluted until the absorbance at 732 nm became 0.7. Adjusted ABTS solution was reacted with mTN followed by a 30-min incubation at RT. Absorbance of the solution was measured at 732 nm via a microplate reader. 0.5 mg/ml of ascorbic acid solution was used as the 100% standard for Fe conversion as well as ABTS inhibition assays. For the DCF-DA assay, 20,000 cells hADSCs were seeded in a 24-well tissue culture plates followed by 24-h incubation. After incubation, the culture medium was removed and 100 μl of DCF-DA solution (25 μM) was added for 45 min under standard culture conditions. Then, the DCF-DA solution was replaced with media with or without 400-μM H_2_O_2_ and 2-μg/ml mTNs. After incubation, the fluorescence of cells was measured via microplate reader and observed via fluorescence microscopy (excitation: 485 nm/emission: 535 nm).

### Anti-inflammatory effect of mTNs in a peritonitis mouse model

Peritonitis mouse experiments were approved by the POSTECH Biotech Center Ethics Committee under guidelines and regulations provided by the POSTECH Institutional Animal Care and Use Committee (IACUC, POSTECH-2019-0021). BALB/c mice were obtained from Joong ah Bio (Suwon, Korea). BALB/c mice (4 weeks old) were treated with 800 μl of 1-mg/ml zymosan to induce peritonitis. After 1 h, 40 μM [TA], mTNs (corresponding to 40 μM [TA]), or DW was injected into mice (intraperitoneal injection). After 5 h, peritoneal fluids were extracted from the zymosan-induced model (< 1.5 ml) and then blood was obtained. To investigate the anti-inflammatory effects of TMP, IL-6 and TNF-α levels in peritoneal fluid were evaluated by ELISA following the assay protocols of the manufacturers.

### Effect of mTNs on osteogenesis

hADSCs (20,000 cells) were cultured in an osteogenic differentiation medium (ODM) (LG-DMEM supplemented with 10% FBS, 1% PS, 50 μg/ml ascorbic acid, 0.01 M of glycerol-2-phosphate, and 100 nM dexamethasone). In the mTN-treated group, 2 μg/ml of mTNs were mixed with the medium, and the medium was refreshed every 2 days. After 14 days of culture, cells were fixed in 4% paraformaldehyde and stained using alizarin red S solution to visualize the secreted calcium. Stained samples were then quantified by measuring absorbance at 550 nm using an extraction solution (cetlypyridium chloride 100 μg/ml, Na_2_HPO_4_ 10 mM). For the ALP assay, differentiated cells were lysed with Pierce™ RIPA buffer (Thermo Fisher Scientific, Waltham, UK) on Day 7. Then, the lysate was reacted with pNPP for 30 min at 37 °C, and 3 N NaOH was added to stop the reaction. Absorbance of the solution was measured at 405 nm via a microplate reader. hADSCs were cultured for 14 days in ODM with or without 2 μg/ml mTNs and 200 μM H_2_O_2_ to investigate the effect of ROS scavenging of mTNs on the osteogenesis of hADSCs. Samples were lysed with RLT buffer, and mRNA was purified using an RNeasy Mini Kit (Qiagen, Valencia, USA). Then, cDNA was synthesized using Maxime RT Premix, and real-time polymerase chain reactions (RT-PCR) were performed using a StepOnePlus Real-Time PCR System (Applied Biosystems, Foster City, USA) with 40 cycles of melting at 95 °C for 15 s and annealing and extension at 60 °C for 50 s. Comparative threshold cycle (Ct) values were used for the analysis and normalized against *glyceraldehyde-3-phosphate dehydrogenase* (*GAPDH*) expression. The following primers were used: Fw: 5′-CAA GGC TGT GGG CAA GGT-3′, Rv: 5′-GGA AGG CCA TGC CAG TGA-3′ (*GAPDH*); Fw: 5′-GCA GTT CCC AAG CAT TTC AT-3′, Rv: 5′-CACTCT GGC TTT GGG AAG AG-3′ (*RUNX2*); Fw: 5′-TGA AAC GAG TCA GCT GGA TG-3′, Rv: 5′-TGA AAT TCA TGG CTG TGG AA-3′ (*OPN*); Fw: 5′-GTG CAG AGT CCA GCA AAG GT-3′, Rv: 5′-TCA GCC AAC TCG TCA CAG TC-3′ (*OCN*).

### Statistical analysis

All quantitative data are presented as means ± standard deviations. Statistical significance was assessed using the paired Student’s t-test and one-way analysis of variance (ANOVA) with post-hoc testing using Tukey’s honestly significant difference test, all performed using GraphPad Prism 7 software (La Jolla, CA, USA). *p*-values < 0.05 were considered statistically significant.

## Results

**Scheme 1:**
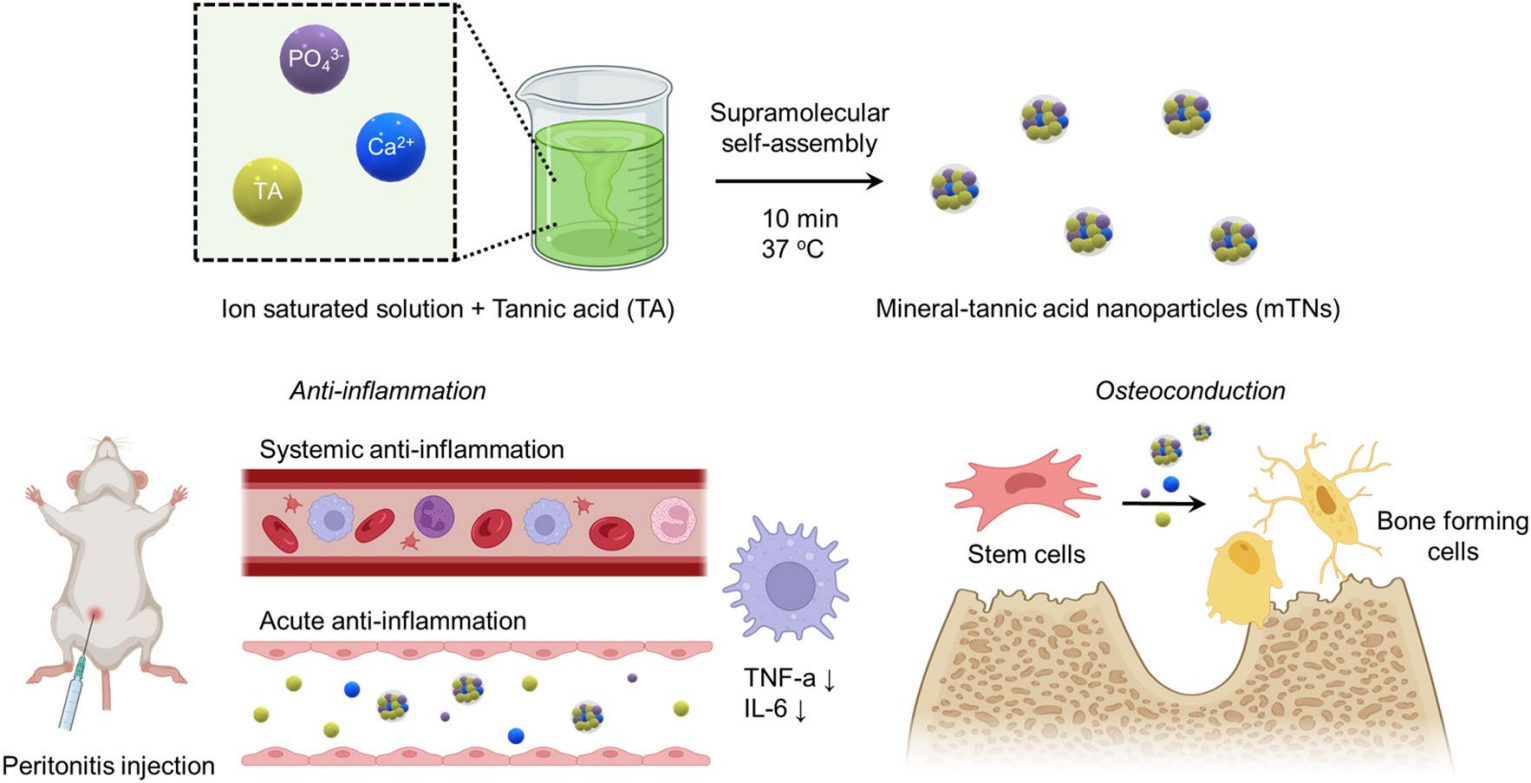
Schematic illustration of mTN fabrication and multi-functionality of mTNs

The schematic illustration of this study is presented in Scheme 1. We fabricated mineral-tannic acid nanoparticles by mixing tannic acid and ion saturated solution via supramolecular self-assembly. The prepared nanoparticles demonstrated strong ROS scavenging capacity and anti-inflammatory effects. The osteoconductivity of the nanoparticles was confirmed through osteogenic differentiation of hADSCs under a highly oxidative environment.

### Fabrication of nanoparticles

**Fig. 1:**
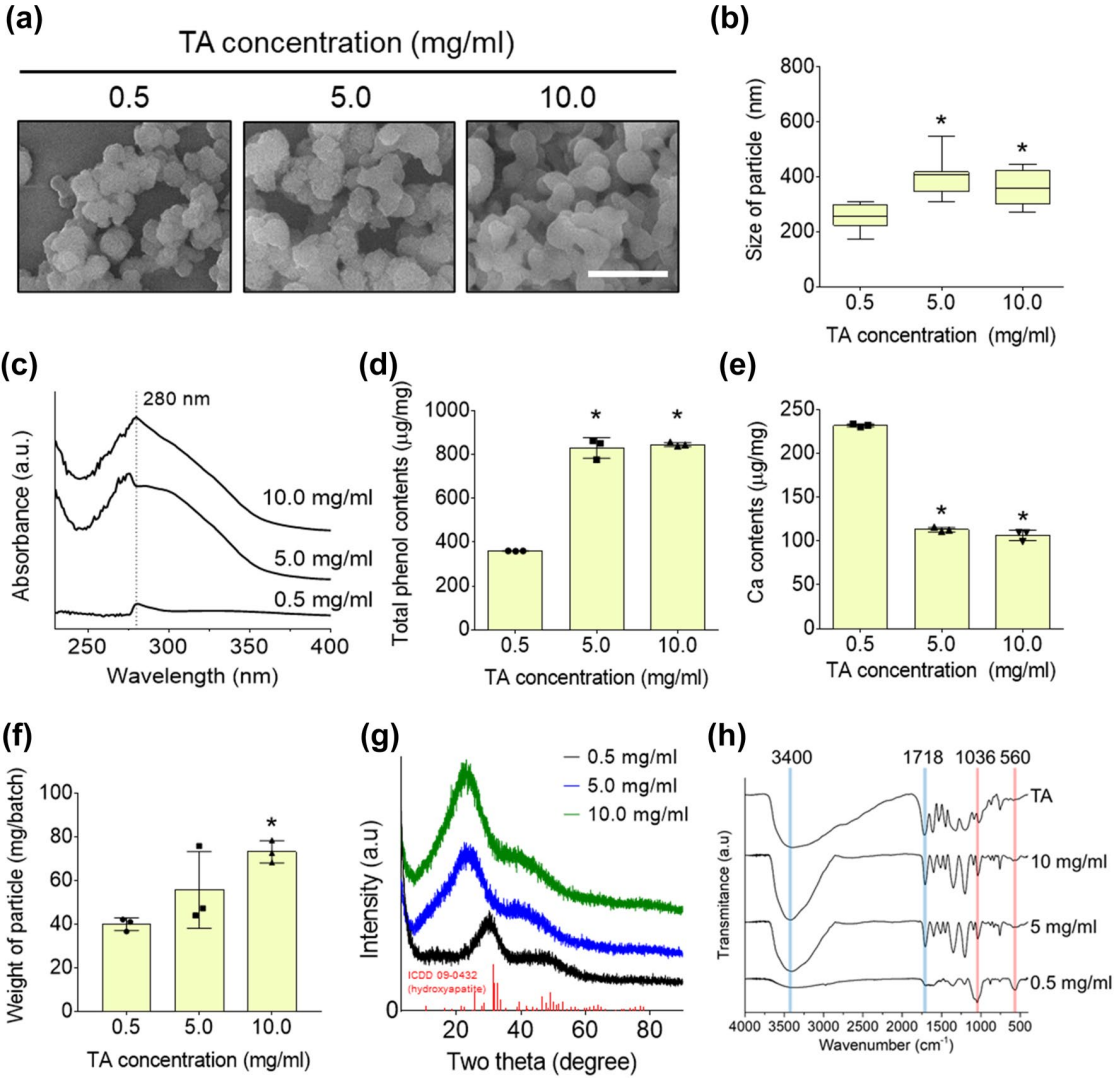
**a** SEM images and **b** corresponding sizes of mTNs prepared at various concentrations of TA (scale bar = 1 μm). ∗Significantly different compared to 0.5 mg/ml of TA (*p* < 0.05). **c** Absorbance spectra of mTNs dispersed in DW. **d** Total phenol content and **e** calcium content of mTNs prepared with various concentrations of TA. ∗Significantly different compared to 0.5 mg/ml (*p* < 0.05). **f** Weight of mTNs prepared at various TA concentrations. ∗Significantly different compared to 0.5 mg/ml (*p* < 0.05). **g** XRD patterns of mTNs and commercially available hydroxyapatite. **h** FT-IR analysis of TA and mTNs

SEM images of mTNs prepared using various concentrations of TA showed spherical shapes in all groups (Fig. 1a). Furthermore, nanoparticles had a homogeneous size distribution that increased slightly as the TA concentration increased (0.5 mg/ml TA: 256 ± 43, 5.0 mg/ml: 393 ± 58, 10.0 mg/ml TA: 358 ± 62 μm) (Fig. 1b). The absorbance of the mTN suspension had a distinct peak at 280 nm in all groups and mTNs prepared using 5.0 and 10.0 mg/ml TA showed a dramatic increase in peak intensity (Fig. 1c). Total phenol content of the mTNs was saturated when > 5.0 mg/ml TA was used and the corresponding calcium content decreased in those groups (Fig. 1d and e). The weight of prepared nanoparticles (lyophilized) increased in proportion to the concentration of TA (0.5 mg/ml: 40 ± 3, 5.0 mg/ml: 56 ± 18, 10.0 mg/ml: 73 ± 5 μg/batch) (Fig. 1f). XRD results showed distinct TA specific peaks at 20° in mTNs prepared from 5.0 and 10.0 mg/ml TA while a hydroxyapatite-like peak around 30° was found in mTNs obtained from 0.5 mg/ml TA (Fig. 1g). FT-IR results revealed that TA and mTNs prepared using 5.0 and 10.0 mg/ml TA showed an OH stretch from 3000 to 3700 cm^−1^ [33] and a keto C=O bond at 1,718 cm^−1^ [34], while those prepared using 0.5 mg/ml TA showed peaks at 560 and 1036 cm^−1^ [35], which are phosphate-specific peaks (Fig. 1h).

### Chemical analysis of supramolecular self-assembly of mTNs

**Fig. 2:**
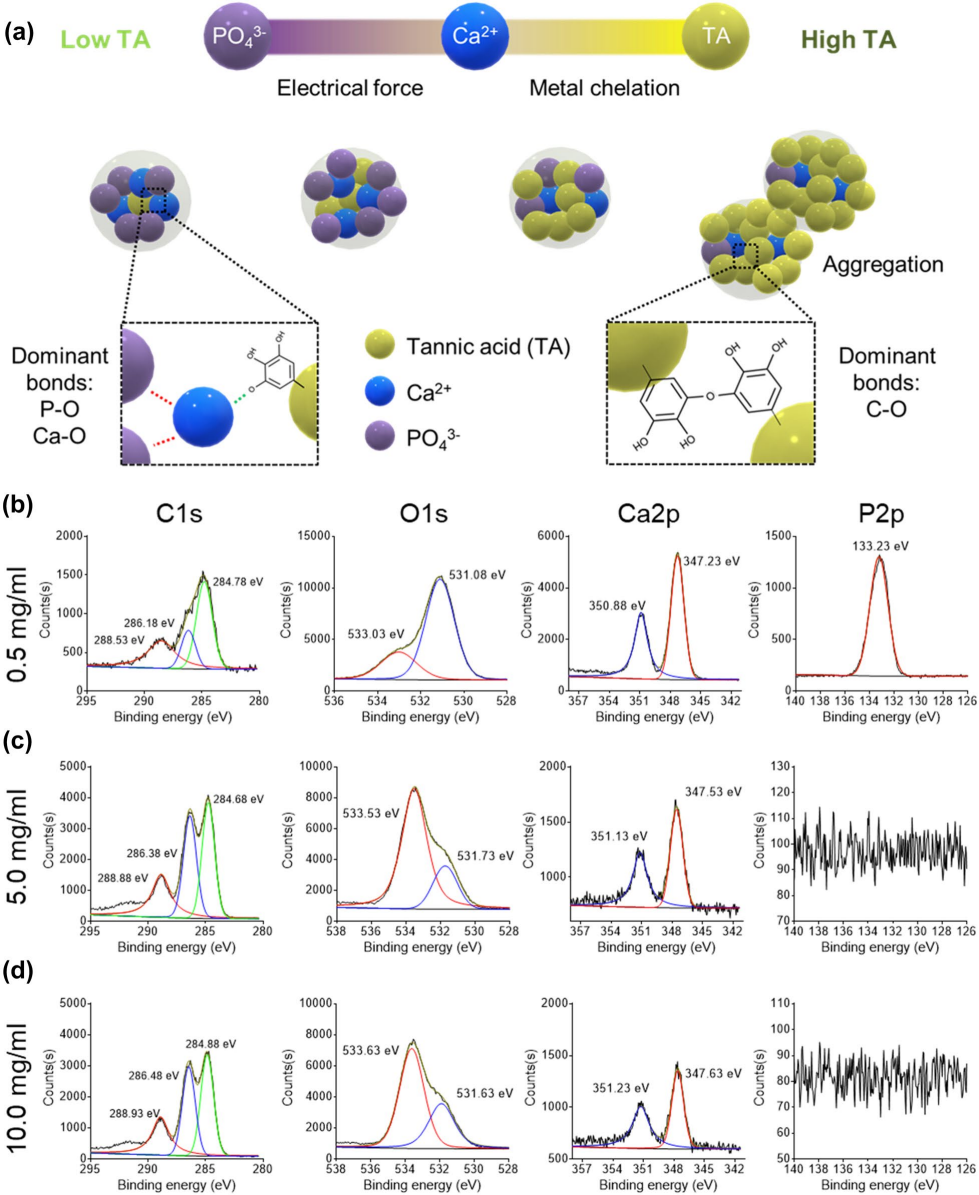
**a** Schematic illustration of supramolecular self-assembly of mTNs. High-resolution **b** C1s, **c** O1s, **d** Ca2p, and **e** P2p XPS spectra of mTNs prepared using various concentrations of TA

A schematic illustration of the proposed mTN assembly process is provided in Fig. 2a. Briefly, electrical attractive forces between Ca^2+^ and PO_4_^3−^ are likely dominant under lower TA conditions while metal chelation between Ca^2+^ and TA likely increases under higher TA conditions, resulting in aggregation of particles owing to hydrogen bond formation. High-resolution XPS results for C1s showed less noise in mTNs prepared from 5.0 and 10.0 mg/ml TA than 0.5 mg/ml TA, implying the presence of more organic compounds. Furthermore, mTNs prepared from 5.0 and 10.0 mg/ml TA showed more C–O–C bonds (~ 286 eV) [36] than observed for the 0.5 mg/ml group (Fig. 2b). High-resolution O1s XPS results showed a peak shift from 531 eV (metal oxides or metal carbonates) [37] to 533 eV (organic C–O) [38] as the TA concentration increased, indicating the presence of a large number of organic bonds in mTNs prepared from 5.0 and 10.0 mg/ml TA (Fig. 2c). Distinct peaks around 350 eV and 347 eV for Ca2p [39] were present in all groups and less noise was detected in the 0.5 mg/ml group than the other groups (Fig. 2d). In the P2p region, a peak at 133.23 eV [40] was found only in mTNs prepared from 0.5 mg/ml TA, representing P-O bonds (Fig. 2e).

### Cytocompatibility of mTNs

**Fig. 3:**
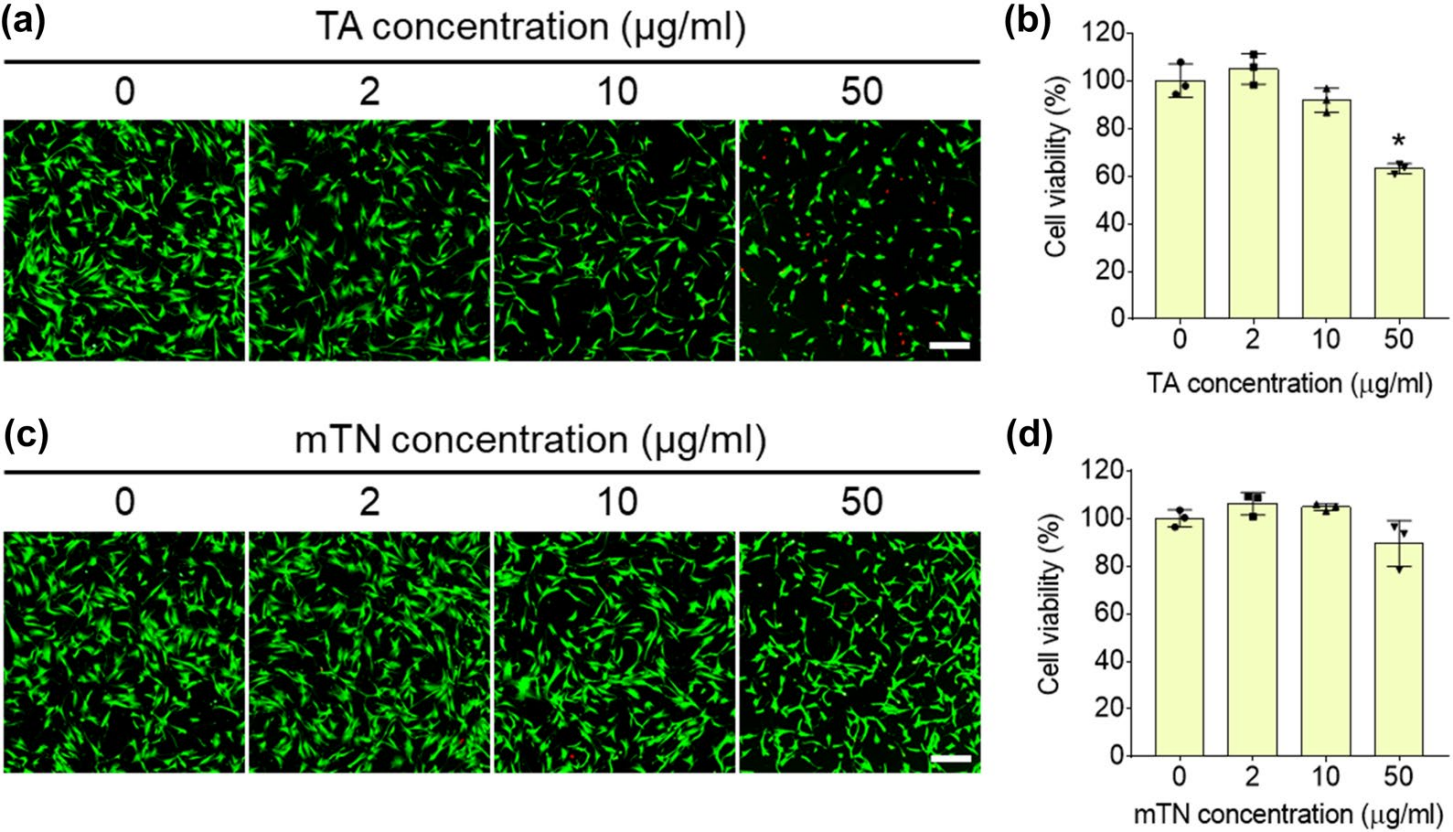
**a** Live/dead images and **b** MTT assay results of hADSCs cultured using various concentrations of TA (scale bar = 200 μm). ∗Significantly different compared to the group with no nanoparticles (*p* < 0.05). **c** Live/dead images and **d** MTT assay results of hADSCs cultured using various concentrations of mTNs (scale bar = 200 μm). ∗Significantly different compared to the group with no nanoparticles (*p* < 0.05)

Live/dead images of hADSCs treated with TA demonstrated that cell spreading was similar in all groups, while the group treated with 50 μg/ml TA had a shrunken morphology and several dead cells were present. These results were consistent with the quantitative MTT assay results, which showed a significant decrease in cell viability in the 50-μg/ml group (0 μg/ml: 100 ± 7; 50 μg/ml: 63 ± 2%) (Fig. 3a and b). Live/dead images of hADSCs treated with mTN showed a similar cell density and spreading area in all groups with no dead cell signals (Fig. 3c). Consistently, the MTT assay results of mTN-treated cells showed similar cell viability in all groups (Fig. 3d).

### ROS scavenging capacity of mTNs

**Fig. 4:**
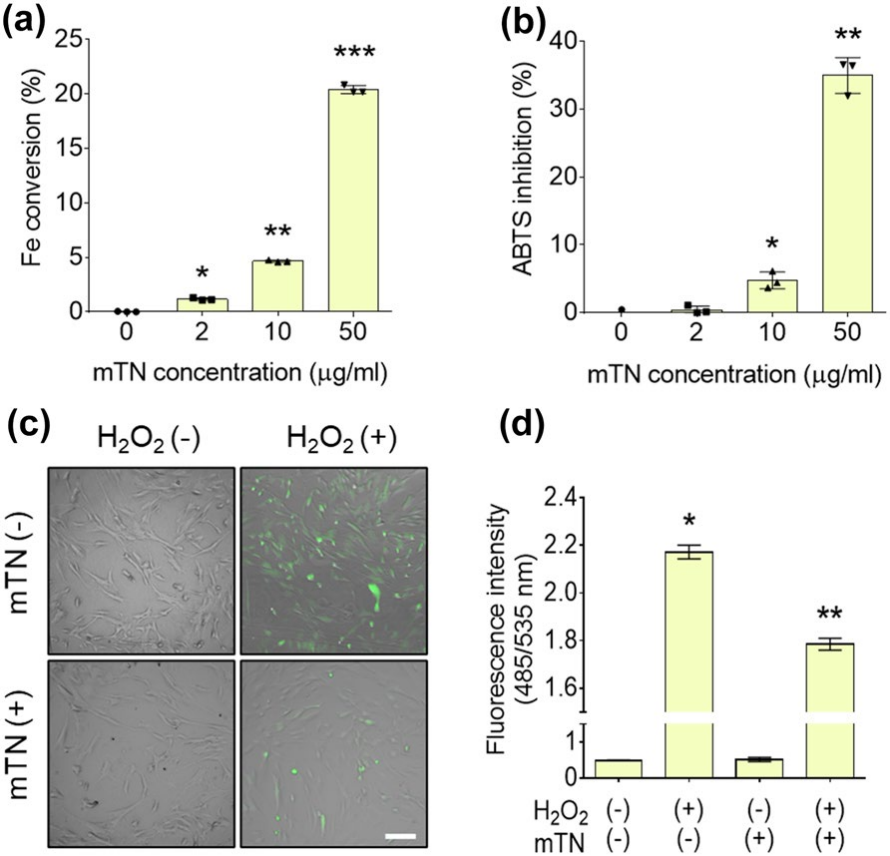
**a** Fe conversion and **b** ABTS inhibition assay results for various concentrations of mTNs. ∗Significantly different compared to the group treated with no nanoparticles (*p* < 0.05). **c** DCF-DA assay images and **d** quantitative analysis of the fluorescence intensity of hADSCs cultured with or without H_2_O_2_ and mTNs (scale bar = 100 μm). *Significantly different compared to the H_2_O_2_ (−)/mTN (−) group (*p* < 0.05)

Fe conversion and ABTS inhibition of mTNs increased in proportion to the concentration of mTNs. Fe conversion was minimal in the absence of mTNs but increased significantly to 20 ± 0% in the presence of 50-μg/ml mTNs. ABTS inhibition increased significantly to 35 ± 3% in the presence of 50-μg/ml mTNs (Fig. 4a and b). Fluorescence images of the DCF-DA assay showed distinct DCF signals in hADSCs treated with H_2_O_2_ but were more intense and brighter in cells cultured without mTNs (Fig. 4c). In the DCF-DA assay, fluorescence intensity was significantly higher in cells treated with H_2_O_2_ and decreased as mTNs were co-administered with H_2_O_2_ [H_2_O_2_ (−)/mTN (−): 0.49 ± 0.01, H_2_O_2_ (+)/mTN (−): 2.17 ± 0.02, H_2_O_2_ (−)/mTN (+): 0.52 ± 0.06, H_2_O_2_ (+)/mTN (+): 1.79 ± 0.02] (Fig. 4d).

### Anti-inflammatory effect of mTNs

**Fig. 5:**
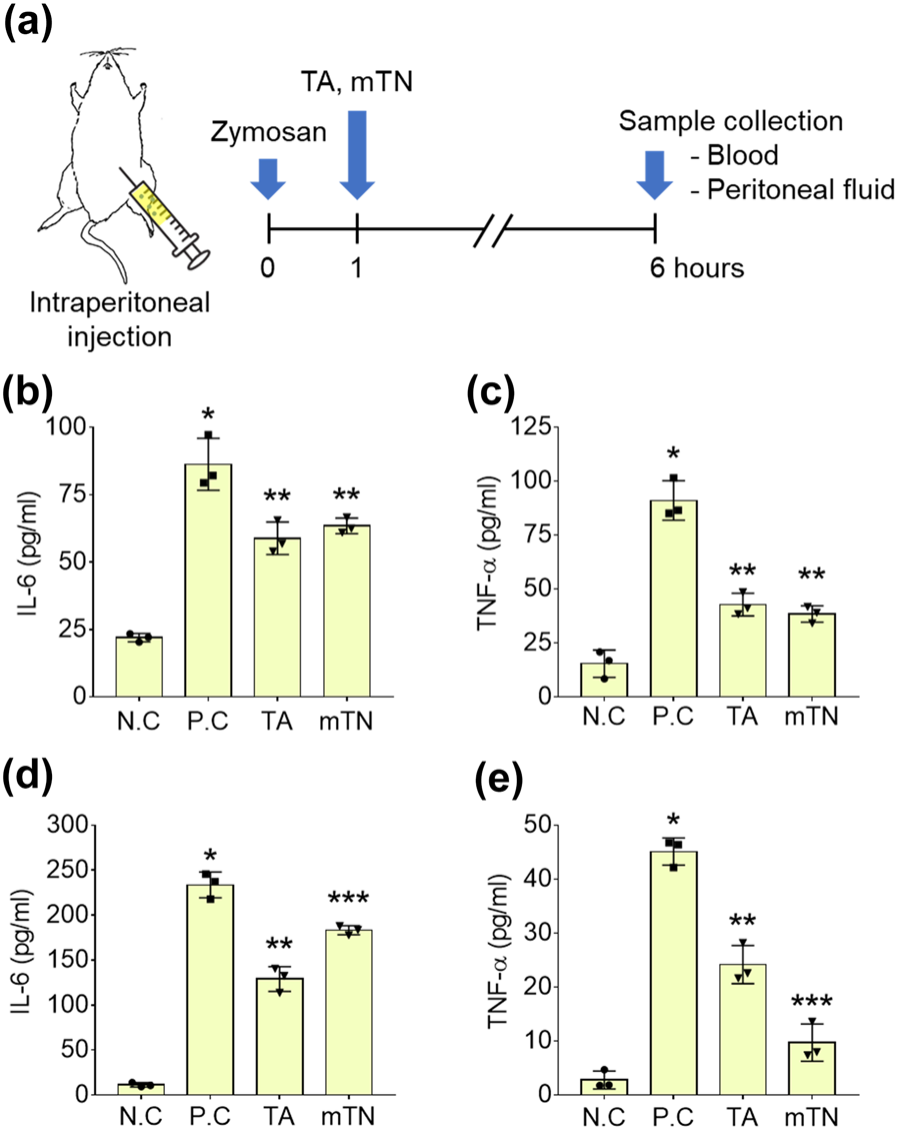
**a** Schematic illustration of the anti-inflammation activity of mTNs in a mouse peritonitis model and the experimental timeline. ELISA results for (**b**) IL-6 and (**c**) TNF-α in the blood. *Significantly different compared to N.C. (*p* < 0.05). ELISA results for (**d**) IL-6 and (**e**) TNF-α in the peritoneal fluid. N.C mice were treated with DW only while the P.C group was treated with zymosan only. *Significantly different compared to the N.C group (*p* < 0.05)

A schematic illustration of the anti-inflammatory assay in a mouse peritonitis model and a timeline is presented in Fig. 5a. Zymosan was injected into mice via intraperitoneal injection followed by TA and mTNs 1 d after induction of inflammation. Blood and peritoneal fluid were obtained after 6 h of treatment. IL-6 level in the blood was increased in all groups compared to the negative control (N.C; DW treatment); however, less IL-6 was detected in mice injected with TA and mTNs than in the positive control (P.C: zymosan treatment) (N.C: 22 ± 2, P.C: 86 ± 10, TA: 59 ± 6, mTNs: 63 ± 3 pg/ml) (Fig. 5b). TNF-α level in blood showed the same trend as IL-6 (N.C: 15.4 ± 6.3, P.C: 91.0 ± 9.1, TA: 42.8 ± 5.3, mTNs: 38.4 ± 3.8 pg/ml) (Fig. 5c). IL-6 in peritoneal fluid in the P.C group was significantly higher than that in the other groups. However, groups treated with TA and mTNs had significantly lower IL-6 levels than those in the P.C group (N.C.: 11.3 ± 2.3, P.C: 237 ± 14, TA: 129 ± 14, mTNs: 183 ± 5 pg/ml) (Fig. 5d). TNF-α levels in the peritoneal fluid of the P.C group were also significantly higher than in the TA and mTN groups. TNF-α levels in the mTN group were lower than those in the TA group (N.C: 3 ± 2, P.C: 45 ± 3, TA: 24 ± 4, mTNs: 10 ± 3 pg/ml) (Fig. 5e).

### Effect of mTNs on the osteogenic differentiation of hADSCs

**Fig. 6:**
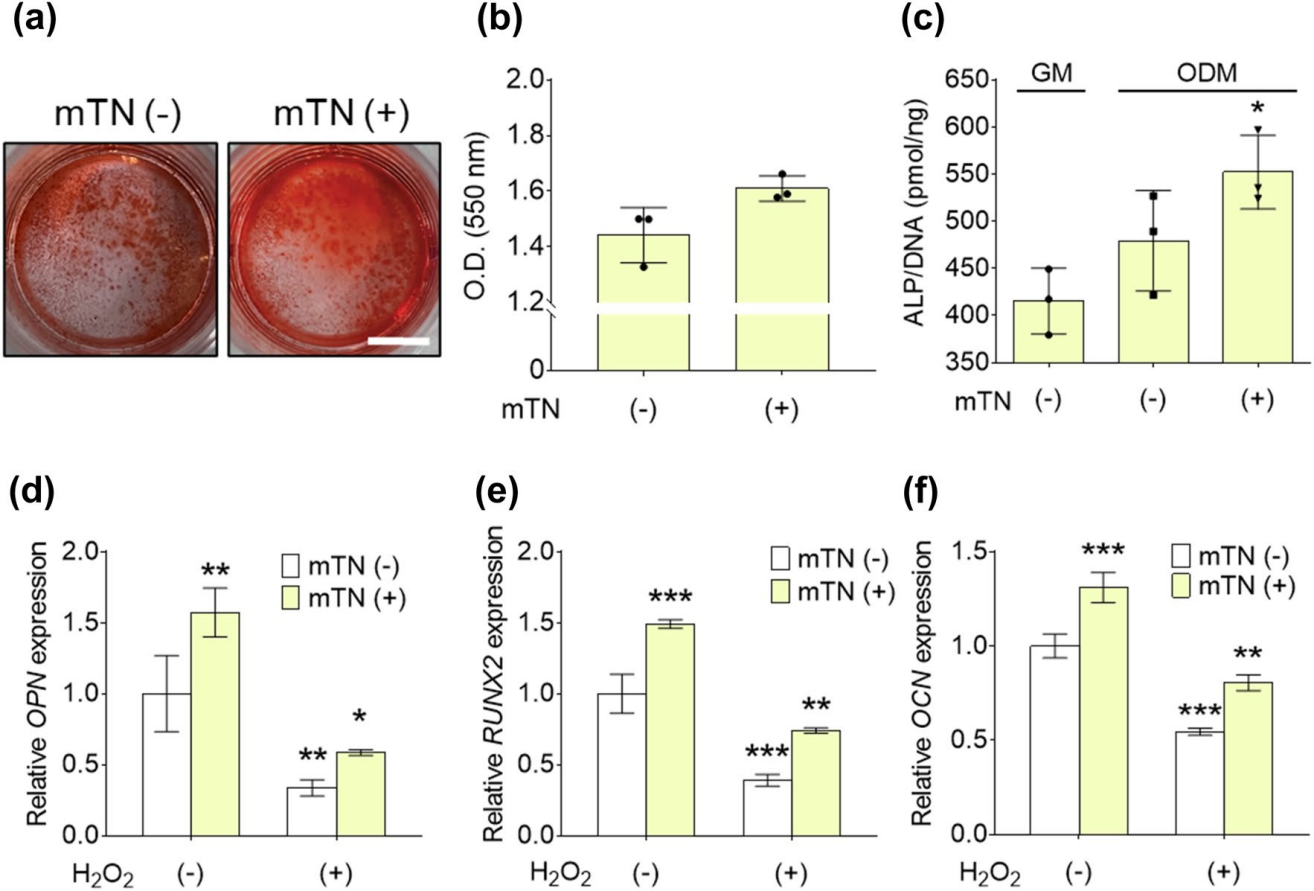
**a** Alizarin red S staining images of hADSCs cultured with or without mTNs for 14 days (scale bar = 5 mm) and **b** quantitative analysis. **c** ALP activity of hADSCs cultured with GM and ODM with or without mTNs for 7 days. *Significantly different compared to GM (*p* < 0.05). Relative mRNA expression of the osteogenic markers (**d**) *OPN*, (**e**) *RUNX2*, and (**f**) *OCN* in hADSCs cultured in ODM with or without H_2_O_2_ and mTNs for 14 days. ∗, ∗ ∗, ∗ ∗ ∗significantly different compared to H_2_O_2_ (−)/mTN (−) (∗*p* < 0.033, ∗ ∗*p* < 0.002, ∗ ∗ ∗*p* < 0.001)

Alizarin red S staining revealed more intense red signals in the mTN-treated groups than the groups without mTNs (Fig. 6a). There was no significant difference in absorbance between the groups treated with mTNs and not treated with mTNs [mTN (−): 1 ± 0, mTN (+): 2 ± 0] (Fig. 6b). ALP activity increased upon ODM treatment; however, only the mTN-treated group had significantly higher ALP activity than the other groups on Day 7 [GM/mTN (−): 415 ± 35, ODM/mTN (−): 479 ± 53, ODM/mTN (+): 552 ± 39 pmol/ng DNA] (Fig. 6c). mRNA expression of the osteogenic markers *OPN*, *RUNX2,* and *OCN* was significantly higher in the mTN-treated group than the control group and decreased upon H_2_O_2_ treatment. Gene expression was significantly higher in the mTN-treated group in the presence of H_2_O_2_ (Fig. 6d–f).

## Discussion

TA is widely used for nanoparticle fabrication via self-assembly with metal ions as it possesses a large number of amphiphilic pyrogallol groups that favor the production of colloidal nanoparticles [16]. In this study, we used an ion saturated solution (10 × SBF) to induce metal coordination for initiation of supramolecular self-assembly of TA. The resulting nanoparticles were spherical in shape and had a controlled size distribution (Fig. 1a and b). In addition, as the TA concentration increased, the amount of TA involved in supramolecular self-assembly increased, leading to an increase in the size and total weight of nanoparticles (Fig. 1b–d, f). Supramolecular self-assembly generally follows the LaMer mechanism, wherein under high TA conditions, the increased amount of free monomers results in an increase in the number of nuclei responsible for nanoparticle growth as well as the corresponding weight of the fabricated nanoparticles [41]. Similarly, by using TA and green fluorescence protein (GFP), Shin et al. confirmed that larger particle size was achieved when a high stoichiometric ratio of TA was present during self-assembly [21]. mTNs fabricated with 5.0 and 10.0 mg/ml TA had a TA-specific absorbance peak at 280 nm [42] and TA deposition on nanoparticles was more than doubled compared to when 0.5 mg/ml TA was used (Fig. 1c and d). However, Ca deposition was reduced by almost half, suggesting competitive assembly of organic TA and inorganic ions within nanoparticles (Fig. 1e). An increase in inorganic components also affects the crystallinity of particles. High-crystallinity mineral particles have long-term storage advantages because of their low dissolution rate; however, as most mineral-organic composite materials act through ion release, amorphous minerals are also favored [43]. Shifting of the XRD peak of mTNs with higher TA concentrations to 20° and attenuation of hydroxyapatite specific peaks indicated that the nanoparticles were amorphous (Fig. 1g) [44]. The FT-IR results were consistent with these findings. Phosphate peaks at 1036 and 560 cm^−1^ were observed in all groups except for the TA itself and were more distinct in mTNs at lower TA concentrations. Furthermore, a distinct broad OH peak and the C=O peak in mTNs with higher TA concentrations demonstrated that chemical composition can be controlled by TA concentration (Fig. 1h) [13, 45]. Collectively, our results suggest that the size and inorganic/organic compositions of nanoparticles can be modulated by controlling the TA concentration.

The mechanism of fabrication of mTNs were inferred through chemical analysis. First, the reduced noise of the high-resolution C1s spectrum, the increase in peaks at 286 eV (C–O–C) and 533 eV (organic C–O) in the O1s spectrum, and the decrease in the peak at 531 eV (metal oxide or metal carbonate) in the C1s spectrum under high TA concentrations suggests that interactions between organic TA were dominant (Fig. 2b and c) [46]. At TA concentrations up to 10 mg/ml, nanoparticles that reach the critical limit of super-saturation by the LaMer nucleation mechanism exist as individual particles owing to Coulomb repulsion [17]. As a result, mTNs fabricated with 5- and 10-mg/ml TA showed narrower size distribution as compared to mTNs fabricated with 0.5 mg/ml TA (Additional file 1: Fig. S1). However, aggregation of nanoparticles occurred during coalescence at higher TA concentrations owing to excessive hydrogen bond formation between the abundant TA molecules present in the solution (data not shown). Conversely, the stoichiometric ratio of Ca increased at lower TA concentrations and electrostatic interactions were dominant, leading to an increase in Ca-O and P-O bonds (Fig. 2d and e). Taken together, these results indicate that the mTN fabrication process described here can be used to generate various organic–inorganic composite nanoparticles because the interactions between and amount of TA and minerals in the nanoparticles can be easily controlled.

Heavy metal ions such as Fe, Ag, and Cu have previously been used for rapid supramolecular self-assembly reactions of TA [20, 45, 47–49]. However, metal ions were used only as assembly mediators in these studies, and thus the biological functions of these ions were not fully exploited. Furthermore, the use of ion-saturated solution (10 × SBF) has several advantages. First, an additional stabilization step is required when manufacturing nanoparticles through self-assembly using a single ionic solution given the high solubility of TA-based nanoparticles in water and their pH-sensitivity, leading to instability on hydrophilic substrates [50]. The mTNs in this study maintained their structure in media without an additional stabilization step. We expect that it will be easy to prepare nanoparticles using various functional metal ions such as Mg, Sr, and Ca by changing the composition of the 10 × SBF. Another advantage of using a multi-ionic solution is the presence of a large amount of sodium ions. Sodium salts in a hydrated state can increase colloidal stability by helping repel anionic nanoparticles owing to their kosmotropic property, which may have contributed to the formation of spherical nanoparticles with controlled sizes as shown in Fig. 1a and b [51].

Analysis of cytotoxicity is essential to determine if biomaterials are suitable for therapeutic applications. Despite the strong antioxidation and antibacterial functions of TA, dose- and cell-type dependent cytotoxicity of TA has also been reported. For example, 10-mM TA was found to be cytotoxic to fibroblasts [52] and TA at 8.9 μM increased intracellular ROS and caused human embryonic kidney cell death [53]. Studies were conducted to determine if control of the local concentration of TA would help prevent cytotoxicity. Saowalak et al. found that TA was rarely toxic when in nanoparticle form [54]. However, the cytotoxicity of TA towards hADSCs has not yet been reported. In the current study, mTNs were not toxic to hADSCs, and the dose-dependent ROS scavenging activity of mTNs indicates that they are suitable for use as antioxidative biomaterials (Figs. 3a, 4a, and b). ROS generated by metabolism or infection can induce cell death through DNA damage or mitochondria dysfunction or delay tissue regeneration by inducing inflammation. Therefore, engineering approaches to reduce ROS for tissue regeneration have been evaluated [55]. Generally, the antioxidative effect of polyphenols is due to their phenol groups. Residual phenol groups in nanoparticles that do not participate in oxidation are capable of ROS scavenging, and reacted phenol groups transform into quinones and display antioxidative activity when the pH is changed [1]. In this study, mTNs also regulated intracellular ROS production by hADSCs under an H_2_O_2_-induced high oxidative stress environment (Fig. 4c and d). Polyphenols such as TA directly transform H_2_O_2_ into H_2_O and upregulate intracellular antioxidative enzymes such as superoxide dismutase 1 (SOD-1) and heme oxygenase 1 (HO-1) to reduce intracellular ROS concentrations [6, 56]. Therefore, TA release or TA on the surfaces of nanoparticles may account for the ROS scavenging capacity of mTNs. TA also has strong anti-inflammatory effects. To assess the anti-inflammatory effect of mTNs, we employed a zymosan-induced acute peritonitis (ZIP) model. Zymosan is a polysaccharide derived from bacterial membranes that can induce inflammation by inducing immune cell recruitment into the peritoneal cavity [57]. A previous study demonstrated that TA controlled inflammation by directly scavenging local ROS [49] and reacting with metabolites in vivo [58]. Our results showed that treatment with mTNs downregulated levels of the pro-inflammatory cytokines IL-6 and TNF-α in peritoneal fluid and blood to a level similar to that in mice treated with TA, suggesting that mTNs can control local and systemic inflammation in vivo (Fig. 5).

We hypothesized that mTNs would promote osteogenesis of stem cells because of the presence of Ca^2+^ and PO_4_^3−^ within the nanoparticles. As bone comprises more than 60% inorganic components, inorganic biomaterials such as ceramics and alloys have been widely used in bone tissue engineering [27]. A widely-used inorganic biomaterial is calcium phosphate (CaP)-based hydroxyapatite as it has a chemical composition similar to that of bone [23]. CaP-based biomaterials not only exhibit osteoconductive features but also affect bone formation by regulating calcium signaling and/or adenosine signaling through dissolved Ca^2+^ or PO_4_^3−^ [59]. However, the fabrication of mineral nanoparticles generally requires sintering processes involving high temperatures and pressures for stability and consequently, incorporation or addition of organic materials to nanoparticles is difficult [60, 61]. In contrast, we demonstrated that mTNs can be prepared under physiological conditions, allowing the biological activity of the mineral ions as well as tannic acid to be fully exploited. mTNs increased ALP activity and intracellular calcium deposition in hADSCs (Fig. 6a and b). As phosphorus atoms were rarely found in mTNs as shown in Fig. 2, the ability of the mTNs to enhance the osteogenic differentiation of hADSCs may have been due to a decrease in local ROS or calcium signaling [62]. In addition, ROS is a regulator of bone tissue regeneration and hampers bone induction by down-regulating the expression of genes essential for osteogenesis such as *RUNX2*, *BMP2*, and *ALP* [6, 63]. We demonstrated that mTNs enhanced the osteogenic differentiation of hADSCs in the presence of H_2_O_2_, confirming that protection from ROS-induced oxidative stress is essential for the osteogenesis of hADSCs (Fig. 6d–f). Thus, fabrication of multi-functional mTNs through a simple one-step method is a promising approach for creating biomaterials suitable for bone tissue engineering.

## Conclusions

We developed a one-step method to fabricate multi-functional nanoparticles by using TA and 10 × SBF and inducing supramolecular self-assembly of TA and multiple mineral ions. The size and chemical composition of the mTNs were controlled by changing the concentration of TA during the assembly process and the resulting mTNs were not cytotoxic to hADSCs even at high concentrations, unlike soluble TA at similar concentrations. mTNs showed the ability to scavenge extracellular and intracellular ROS in vitro. mTNs also had an anti-inflammatory effect in a zymosan-induced in vivo mouse peritonitis model. Finally, mTNs enhanced the osteogenic differentiation of hADSCs even under high oxidative stress conditions. Collectively, multi-functional nanoparticles fabricated by supramolecular self-assembly of TA and minerals are useful materials for bone tissue regeneration.

## Supplementary Information

**Additional file 1. Figure S1.** (a) Dynamic light scattering result of mTNs fabricated with different concentrations of tannic acid.

## Data Availability

All data used to generate these results are available in the main text.
