# Supplementary material for: Biomimetic anti-inflammatory and osteogenic nanoparticles self-assembled with mineral ions and tannic acid for tissue engineering
Source: Nano Converg. 2022 Oct 10;9:47. doi: 10.1186/s40580-022-00338-2 (PMC9551158; doi:10.1186/s40580-022-00338-2)
Supplement: Supplementary file 1 — Additional file 1. Figure S1. (a) Dynamic light scattering result of mTNs fabricated with different concentrations of tannic acid. [file 40580_2022_338_MOESM1_ESM.docx]

**Supplementary figure**

Figure S1. (a) Dynamic light scattering result of mTNs fabricated with different concentrations of tannic acid.
